# A miniaturized and integrated dual-channel fluorescence module for multiplex real-time PCR in the portable nucleic acid detection system

**DOI:** 10.3389/fbioe.2022.996456

**Published:** 2022-09-12

**Authors:** Yile Fang, Yue Wang, Xiangyi Su, Haoran Liu, Hui Chen, Zhu Chen, Lian Jin, Nongyue He

**Affiliations:** ^1^ State Key Laboratory of Bioelectronics, School of Biological Science and Medical Engineering, Southeast University, Nanjing, China; ^2^ Economical Forest Cultivation and Utilization of 2011 Collaborative Innovation Center in Hunan Province, Hunan Key Laboratory of Biomedical Nanomaterials and Devices, Hunan University of Technology, Zhuzhou, China

**Keywords:** fluorescence detection, confocal optical path, LED drive circuit, real-time PCR, point-of-care testing

## Abstract

A portable nucleic acid detection (PNAD) system based on real-time polymerase chain reaction (real-time PCR) has been developed for point-of-care testing (POCT) of infectious disease pathogens. In order to achieve “sample-in, result-out” while keeping the system compact, the hardware system integrates optical, thermal and motion control modules in a limited space for nucleic acid extraction, purification, amplification and detection. Among these hardware modules, the fluorescence module is one of the most important modules, because its performance directly affects the accuracy and sensitivity of the testing results. In this paper, a miniaturized, high-sensitivity and integrated dual-channel fluorescence module have been proposed for the homemade PNAD system. Based on the principle of confocal optical path, two group of excitation-emission optical paths of different wavelengths are integrated in a small space. In terms of circuitry, a current-light dual negative feedback light emitting diode (LED) drive circuit is applied to improve the stability of the excited light source. All optical and electronic components are integrated in a metal box of 55 mm × 45 mm × 15 mm, that helps miniaturize the detection system. Two different modules have been assembled to fit various fluorescent dyes or probes with the set of excitation and emission as follow: module 1#: 470 nm/525 nm, 570 nm/630 nm; module 2#: 520 nm/570 nm, 630 nm/690 nm. Finally, hepatitis B virus (HBV) concentration gradient detection and multiplex detection of different gene targets of SARS-CoV-2 are carried out on the PNAD system equipped with these two fluorescence modules for evaluating their performances. Compared with the commercial real-time PCR instrument, our fluorescence module has good stability and detection sensitivity.

## Introduction

Nowadays, the Corona Virus Disease 2019(COVID-19) ravages the world with clinical manifestations ranging from cough and fever to pneumonia and even death, and its high infection and mortality rates bring great inconvenience and loss to people’s lives and the global economy (Bedford et al., 2020; Benmalek et al., 2021). The detection methods for COVID-19 mainly include antigen testing and nucleic acid testing (Cui and Zhou, 2020; Mathuria et al., 2020; Pokhrel et al., 2020; Rai et al., 2021). The antigen test measures some of the viral proteins and indicates the presence of pathogens. Compared with the nucleic acid method, antigen test is simple to perform and has a shorter turnaround time, but its low sensitivity makes unreliable results. Nucleic acid test detects viruses by amplifying the targeted nucleic acids and is used to confirm the presence of viruses that are difficult to culture, making it the gold standard test for COVID-19 microbiological diagnosis (Zhu et al., 2020; Song et al., 2021). But traditional nucleic acid testing is only suitable for large centralized diagnostic laboratories which require multiple devices to complete. The rapid development of COVID-19 and the limited capabilities of laboratory-based molecular detection make point-of-care testing (POCT) urgently needed in the diagnosis of COVID-19 outside the laboratory setting (Li and Ren, 2020; Tang et al., 2020). The greatest merit of POCT is that it could realize fast tests with low cost, providing sufficient time for the implementation of necessary preventive and therapeutic measures (Chen et al., 2018; Hanson et al., 2020; Deng et al., 2021; Huang et al., 2021; Wang et al., 2021).

**FIGURE 1:**
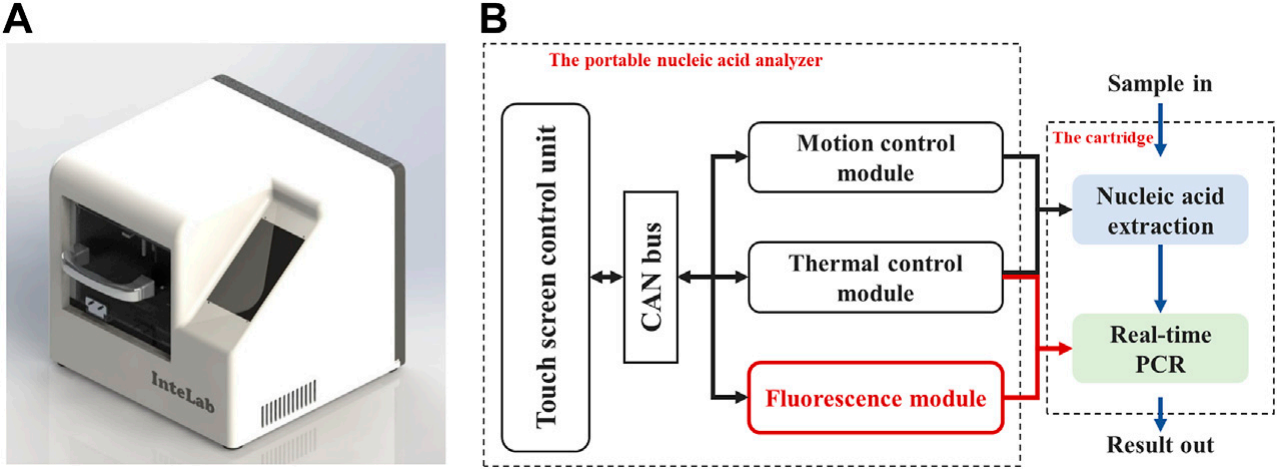
Homemade portable nucleic acid detection (PNAD) system. **(A)** 3D rendering of the PNAD device. **(B)** Functional block diagram of the PNAD system including motion control module, thermal control module and fluorescence module to achieve “sample-in, result-out”.

Based on the above background, our group has developed a portable nucleic acid detection (PNAD) system (Figure 1A), which can automatically perform nucleic acid extraction and real-time PCR without manual intervention to achieve “sample-in, result-out” (Chen et al., 2017; Fang et al., 2021). As shown in Figure 1B, there are three core functional modules in this system including motion control module, thermal control module and fluorescence module, in which the fluorescence module is the crux of real-time PCR, because its sensitivity and stability determine whether real-time PCR can accurately quantify DNA templates. Meanwhile, its structure needs to be as compact as possible for miniaturization and portability.

Fluorescence is a photoluminescence phenomenon. When a substance absorbs incoming light, the energy of the photon is transferred to the molecule. After excitation occurs, the electrons of the molecule jump from a low energy level to a high energy level, i.e., from the ground state to an excited single or excited triplet state. Molecules in excited states are unstable and must be released by radiative transfer or non-radiative transfer to return to the ground state. The energy in the radiative transfer is released as photons (Ishikawa-Ankerhold at al., 2012; Fu et al., 2020). Compared to other optical detection techniques, fluorescence detection has high sensitivity, specificity and accuracy. Besides, fluorescence detection is immune to electromagnetic, pressure and temperature interference, so it also has a high stability. With the development of fluorescence detection technology, portable instruments based on fluorescence are developed and applied in various fields. However, fluorescence modules reported in the literature and used in commercial equipment either only have a single excitation light or a split structure with separate light sources and detectors. For example, Novak et al. (Novak et al., 2007) developed a fluorescence detection module consisting of a blue LED, excitation and detection filters, dichroic mirrors, and photodiodes. Katzmeier et al. (Katzmeier et al., 2019) reported a pocket-sized fluorescence detector for POCT, the detection unit of which includes an excitation light and a photodetector. Both of the above mentioned had only one excitation source and didn’t allow for multiple detection. Alam et al. (Alam et al., 2019) designed a portable fluorometer for detection of breast cancer cells, the excitation source and the detector are placed on opposite sides of the sample in a linear fashion. This separated structure was not favorable to integration.

In this study, a miniaturized, high-sensitivity and integrated dual-channel fluorescence module was designed for special requirements of the homemade PNAD system. The optical path was based on a confocal structure, which enables the excitation and emission of light without interference. Different from split structure, the excitation light source and detector of this module were located on the same side to make the structure more compact. Due to the generally weak fluorescence signal, it is crucial that the light source and detector should be stable and sensitive. A current-light dual negative feedback drive circuit was used to adjust the light intensity of the light emitting diode (LED) for its stability. As for the detection portion, the fluorescence signal was processed by amplification, filtering and analogue-to-digital (A/D) to confirm the accuracy and stability of the fluorescence data. After fabrication, two fluorescence modules containing 4 different fluorescence channels were integrated into the PNAD system, and hepatitis B virus (HBV) concentration gradient detection and multiplex detection of different gene targets of SARS-CoV-2 have been carried out for evaluating the performance of the modules. The results showed that the fluorescence module has excellent has excellent linearity with R2 greater than 0.99 and reproducibility with coefficient of variation (CV) less than 2%. And this fluorescence has good homogeneity of each channels compared with the commercial StepOnePlus.

## Materials and methods

### Architecture of the fluorescence module

**FIGURE 2:**
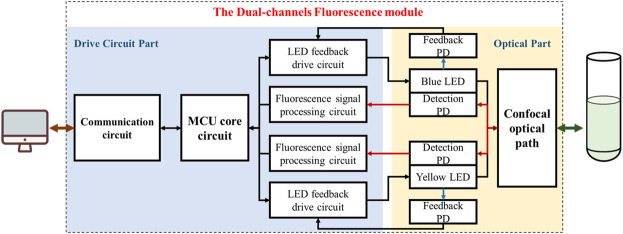
The Functional block diagram of the dual-channel fluorescence module, which can be divided into two partitions of drive circuit and optical path (Take module 1# as an example).

Figure 2 shows the functional block diagram of the fluorescence module. The whole module can be divided into two parts of a drive circuit and an optical path. As for the first part, a STM32 series microcontroller (STM32F103C8T6, STMicroelectronics) was used as the microprogrammed control unit (MCU) of the module, which realized data and commands interaction with outside through controller area network (CAN) bus while controlled the excitation LEDs by outputting signals through the input/output (I/O) ports. A current-light dual negative feedback LED drive circuit was designed to enhance the stability of the excitation light. Moreover, a fluorescence signal processing circuit was designed to perform amplification, filtering and analog-to-digital (A/D) conversion on optoelectronic signals from detection photodiodes (PD). In terms of the optical part, LEDs, PDs and other optical elements such as filters, dichroic mirrors and lens were fixed on the confocal light path. The module integrated two similar excitation/emission optical path and corresponding drive circuits for achieving dual-channel measurement.

The module was executed as follows: First, the MCU controlled the LED to excit the sample through excitation light path. Then, the sample absorbed the specific frequency of photons and emitted the fluorescence. After passing through the emission light path, the fluorescence signal was received by the detection photodiode and converted into electrical signal, which was then processed by the photoelectric signal processing circuit for amplification, filtering, and finally transmitted to the host computer through the communication unit after analog-to-digital (A/D) conversion.

### Optical design and components selection

Common optical systems used for fluorescence detection include light sources, focusing lenses, filters, dichroic mirrors and photodetectors. It is important to select an appropriate excitation light according to the requirements of application, which affects the sensitivity and selectivity of detection. Real-time PCR commonly used excitation light sources are xenon lamps, mercury lamps, lasers and high-power LEDs (Spibey et al., 2001; Shin et al., 2021), different lights have their own applications due to their characteristics. For example, xenon lamps and mercury lamps are applied to high power analysis systems with high luminous intensity, but their large size is not allowed in miniaturized instruments. Lasers have high brightness and good monochromaticity but their prices are expensive. Compared with other lights, LED is more suitable for portable systems with low-cost, small size, low energy consumption, long life, etc. Therefore, we chose high-power monochromatic LEDs of different wavelengths as light sources (LB W5SM, LY W5SM, LT W5SM, LR W5SM, OSRAM GmbH.). The wavelength of these 4 LEDs and corresponding fluorescence dyes are listed in Table 1. As for photoelectric sensors, the mainstream photodetectors include avalanche diode (APD), charge-coupled device (CCD), photomultiplier tube (PMT) and photodiodes (PD). APD has high gain, high quantum efficiency but narrow spectral response range, CCD can scan all fluorescent signals at the same time but with low sensitivity, PMT would be a good choice profits from its fast response and low noise without considering price. In contrast, PD is the most suitable detector in our system because of its high sensitivity, low noise and low-cost (Song et al., 2010; Ryu et al., 2011; Abdallah et al., 2014; Zhan et al., 2019), so the silicon PDs (S1337-33BR, Hamamatsu Photonics Co., Ltd.) were chosen as the detector.

**TABLE 1:** Fluorescence dyes excitation and emission wavelengths.

	LED	Fluorescence dye	Excitation light (nm)	Emission light (nm)
Module 1	LB W5SM	FAM	450–490	510–530
	LY W5SM	Texas Red	560–590	610–650
Module 2	LT W5SM	HEX	515–535	560–580
	LR W5SM	Cy5	620–650	675–690

**FIGURE 3:**
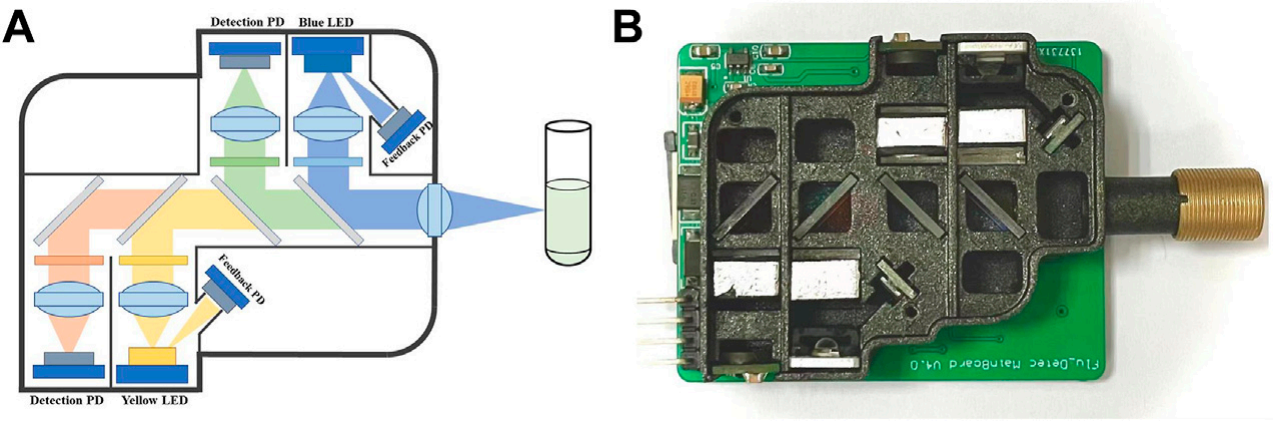
Optical path of the dual-channel fluorescence module (Take module 1# as an example). **(A)** Schematic diagram of optical path: two excitation lights (blue and yellow) and two emitted lights (green and orange) forms a confocal light path with the help of four dichroic mirrors. **(B)** Photograph of the optical path, consistent with the schematic.

To make the module more compact while reducing the interference of external stray light and improving the signal-to-noise ratio (Xie and Yang, 2019; Peng et al., 2021), an optical structure based on confocal light paths was designed as shown in Figure 3A. Figure 3B shows the photograph of the optical path, in which all of the optical elements were installed in a 3D printing box. In order to make the emitted light unaffected by the excitation light, bandpass filters of corresponding wavelengths were set in front of the LEDs and the PDs. In addition, dichroic mirrors in the main optical path were set to separate the lights from each channel.

The bandpass filters and the dichroic mirrors were set as follows:

#### Module 1# channel 1#

The bandpass filter with center wavelength (CWL) of 470 ± 20 nm (BP-470, Shanghai Mega-9 Photoelectric Co., Ltd.) and the dichroic mirror with central wavelength of 500 (DM-500, Shanghai Mega-9 Photoelectric Co., Ltd.) nm were applied for excitation light; The bandpass filter with CWL of 525 ± 20 nm (BP-525) and the dichroic mirror with central wavelength of 550 nm (DM-550) were applied for emission light;

#### Module 1# channel 2#

The bandpass filter with center wavelength (CWL) of 570 ± 20 nm (BP-570) and the dichroic mirror with central wavelength of 600 nm (DM-600) were applied for excitation light; The bandpass filter with CWL of 630 ± 20 nm (BP-630) and the dichroic mirror with central wavelength of 650 (DM-650) nm were adopted for emission light;

#### Module 2# channel 1#

The bandpass filter with center wavelength (CWL) of 520 ± 20 nm (BP-520) and the dichroic mirror with central wavelength of 550 (DM-550) nm were adopted for excitation light; The bandpass filter with CWL of 570 ± 20 nm (BP-570) and the dichroic mirror with central wavelength of 600 nm (DM-600) were adopted for emission light;

#### Module 2# channel 2#

The bandpass filter with center wavelength (CWL) of 630 ± 20 nm (BP-630) and the dichroic mirror with central wavelength of 650 nm (DM-650) were adopted for excitation light; The bandpass filter with CWL of 680 ± 20 nm (BP-680) and the dichroic mirror with central wavelength of 700 (DM-700) nm were adopted for emission light; To achieve light focusing, convex lenses (GL12-006–006, Beijing Golden Way Scientific Co., Ltd.) were also set in front of the LED and the PD, and a focusing lens (GL11-006–008, Beijing Golden Way Scientific Co., Ltd.) was set at the excitation and acquisition ends to make the excitation light focus at the sample.

### The current-light dual negative feedback LED drive circuit

According to the principle of fluorescence detection, the stability of the detected fluorescence signal is affected by the fluctuation of the excitation light. Therefore, a current-light dual negative feedback LED drive circuit was designed to provide stable excitation light.

**FIGURE 4:**
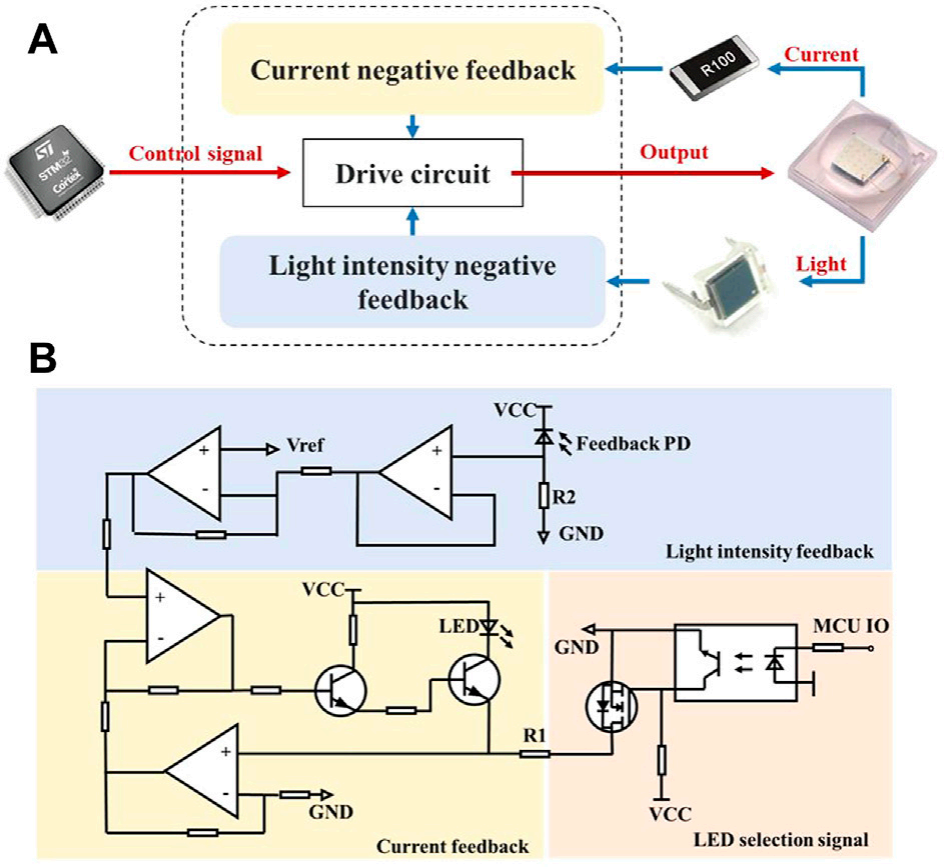
The current-light dual negative feedback LED drive circuit. **(A)** Working schematic block diagram of the circuit, the current-light dual negative feedback is realized by sampling the current and the light intensity of the LED. **(B)** Schematic of the circuit which can be divided into three partitions of current feedback, light intensity feedback and LED selection.

The schematic block diagram is shown in Figure 4A, the current and light intensity adjust the drive circuit together to make the output stable. Due to the negative temperature coefficient of LED, the forward voltage drop will decrease as the temperature rises resulting in an increase of the forward current when driven by a constant voltage source. Meanwhile, a slight change in the forward voltage will cause a large change in the forward current because of volt-ampere non-linearity, so that the LED luminous flux will also be changed. Therefore, constant current source is more appropriate for the LED driver, which can avoid fluctuations in the supply current and thus ensure the stability of the light source. With increased usage time and temperature changes, LED will experience light decay and its light intensity will then diminish. To solve this problem, light intensity negative feedback was added on the basis of current negative feedback.

Figure 4B is the schematic of the drive circuit, which can be divided into three parts: 1) on-off control area: control signal from the MCU controlled LED via a MOSFET (YJL3400A, Yangzhou Yangjie Electronic Technology Co., Ltd.); 2) current feedback area: sampled by a resister R1, LED current was converted to voltage and put into negative feedback, and finally the current was regulated by the output characteristics of a triode (BCP56-16T3G, ON Semiconductor). 3) light intensity feedback area: a feedback PD (BPW 34 BS, OSRAM GmbH.) generated photocurrent when receiving excitation light, which was sampled by a resistor R2 and then connected to feedback terminal. In short, the current negative feedback will increase current when it decreases, and the light intensity negative feedback will increase current to strengthen light when light weakens, and vice versa.

### Fabrication and integration of the module

**FIGURE 5:**
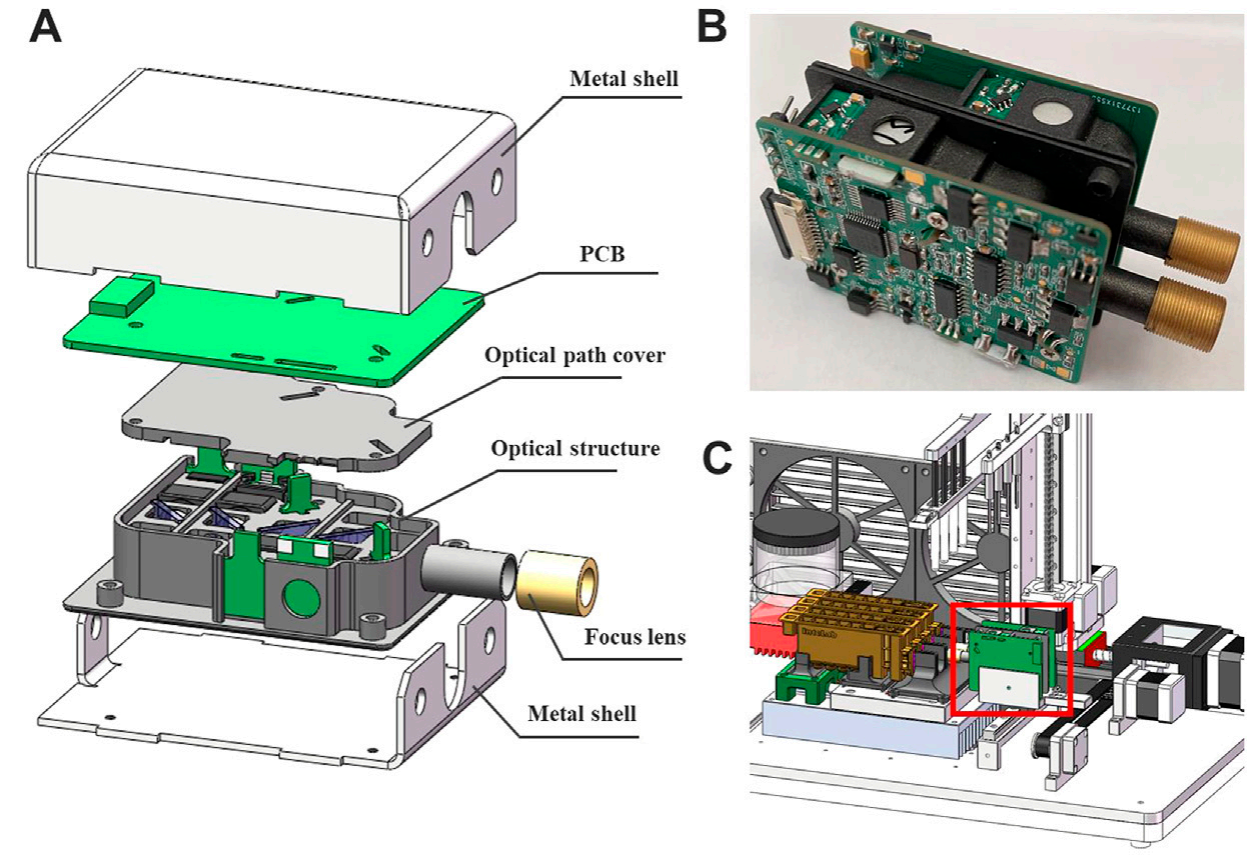
**(A)** The three-dimensional (3D) illustration of the fluorescence module in an exploded view, which includes metal shell, PCB, optical path cover, optical structure and focus lens. **(B)** The photograph of two fluorescence modules combined together. **(C)** Integration of the fluorescence module (in the red box) with a homemade portable nucleic acid detection system.

The exploded view of the overall structure is shown in Figure 5A, all the optical components were placed in a 3D printing box, above which was the control circuit board. The optical path and the circuitry were assembled into a complete unit by soldering the LED board and the PD board to the main circuit board. This assembly was then placed inside a metal box to isolate electromagnetic noise in environment. Two fluorescence modules (Figure 5B) with different wavelengths of excitation light were fabricated and integrated into the PNAD system (Figure 5C) for multiplex real-time PCR. Under the driving of the stepping motor, the fluorescence modules can scan and detect the signal of multiple reaction tubes.

### Reagents and methods for performance evaluation

The two fluorescence modules were installed in the homemade PNAD system after fabrication. Then, HBV and SARS-Cov-2 real-time reverse transcription PCR (real-time RT-PCR) were performed on this system. Meanwhile, the same sample was amplified and detected in StepOnePlus real-time PCR system (Applied Biosystems, America) under the same conditions for reliable results. The reagents used in the experiments include magnetic bead RNA extraction kit (Z-ME-0010, Shanghai Zhijiang Biotechnology Co., Ltd.), hepatitis B virus (HBV) nucleic acid assay kit (Z-HD-2002-02, Shanghai Zhijiang Biotechnology Co., Ltd.), 2019-nCoV nucleic acid test kit (Z-RR-0479-02-50, Shanghai Zhijiang Biotechnology Co., Ltd.) and Sterilized ddH_2_O (Sangon Biotech).

Experimental methods are as follows:

1) Serum samples containing 5 × 10^6^ IU/ml (standard sample in the HBV nucleic acid assay kit) were selected for testing. First, the viral RNA was extracted by the RNA extraction kit. After that, purified HBV RNA was diluted 10×, 100×, 1,000×, and a total 4 concentrations of samples were obtained before and after dilution. Then, 10 μL of each sample was added to 40 μL of the PCR mix, blended and centrifuged. Finally, real-time RT-PCR was performed by the PNAD system and StepOnePlus. The reaction conditions of real-time RT-PCR were: 37°C 2 min, 94°C 2 min, 93°C 15 s 45 cycles and 60°C 60 s. The first channel (excitation: 470 nm and emission: 520 nm) was used to detect the fluorescence signal.
2) SARS-CoV-2 positive control sample of the test kit were extracted by the extraction kit. Then 5 μL of purified RNA and 20 μL of PCR mix were blended to form 25 μL amplification system. Finally, multiplex real-time RT-PCR was performed by the PNAD system and StepOnePlus to test ORFlab gene, N gene, E gene and the internal standard target of SARS-CoV-2. The reaction conditions of real-time RT-PCR were: 45°C 2 min, 95°C 3 min, 95°C 15 s, 45 cycles and 58°C 30 s. The first channel (excitation: 470 nm and emission: 520 nm) was used to detect ORFlab gene, the second channel (excitation: 520 nm and emission: 570 nm) was used to detect N gene, the third channel (excitation: 570 nm and emission: 630 nm) was used to detect E gene, and the fourth channel (excitation: 630 nm and emission: 690 nm) was used to detect the internal standard target.

The fluorescence module designed in this paper was used for fluorescence excitation and detection. It took 200 ms to detect each sample tube, 5 raw fluorescence data were collected and averaged as the final fluorescence value. Finally, the fluorescence values of each cycle were plotted as real-time PCR curves for further analysis.

## Results and discussion

### Optical path simulation results and analysis

**FIGURE 6:**
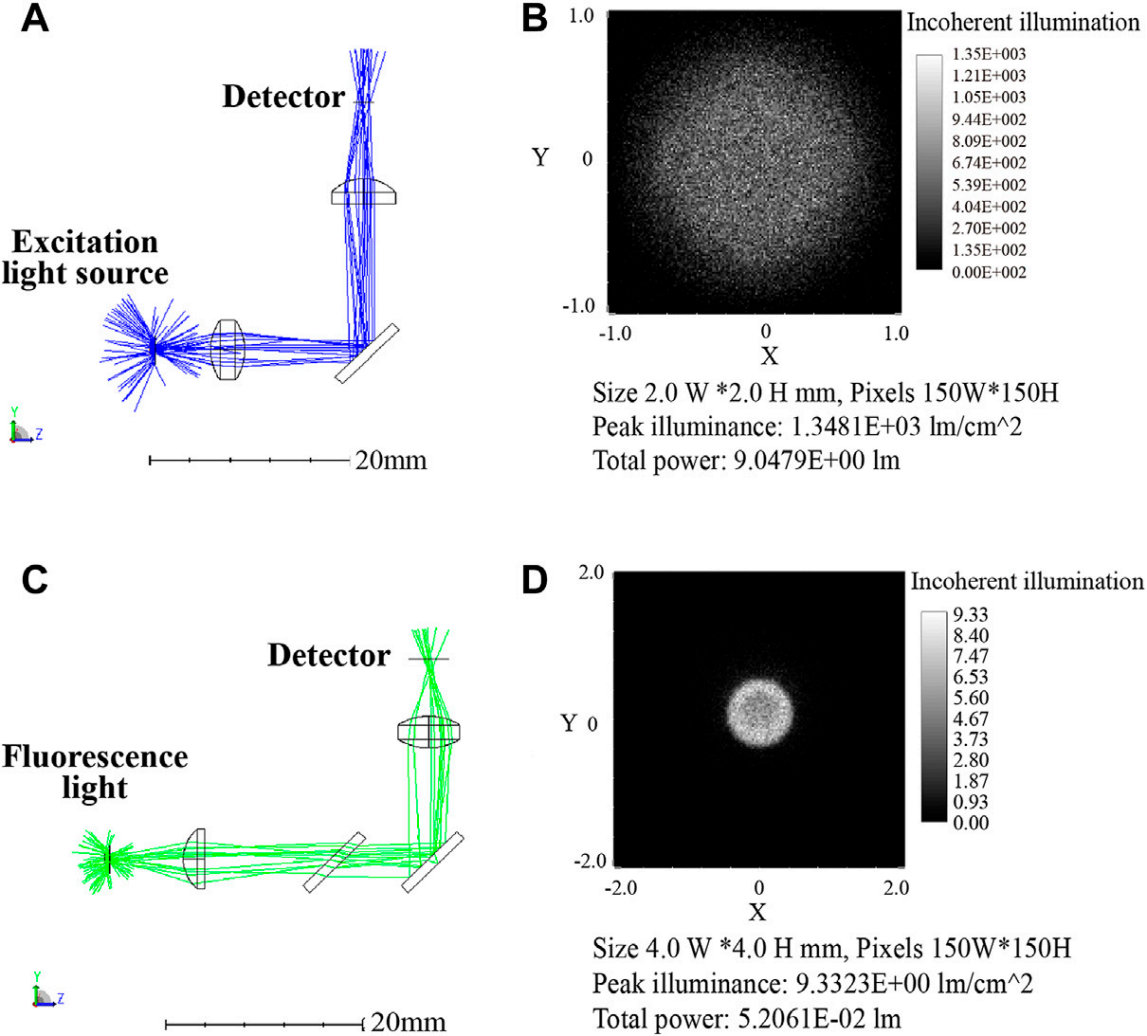
First channel optical paths (excitation/emission: 470 nm/525 nm) simulation diagram **(A)** 3D layout of the excitation optical path. **(B)** Image of the excitation optical path detector with 9.05 l m total power. **(C)** 3D layout of the emission optical path. **(D)** Image of the emission optical path detector with 5.2 × 10^-2^ lm total power.

In order to further analyze the feasibility of the optical path, the transmission efficiency and detector received light intensity were simulated by the ZEMAX software. And the optical paths of the two channels were simulated separately, with each channel divided into two optical paths: excitation and emission. The first channel optical paths simulation diagram is shown in Figure 6, Figure 6A indicates the 3D layout of the excitation optical, where the excitation light was collimated by a focusing lens, reflected by a dichroic mirror, and then focused by a plano-convex lens onto a detector. Figure 6B shows the light spot on the detector that was set up to evaluate the excitation light intensity at the sample. Likewise, Figure 6C presents the 3D layout of the emission light path, compared with the excitation light path, an additional dichroic mirror was set to separate the excitation light from the emission light. Figure 6D shows the light spot on the detector which represented the light received by the PD in the actual module.

Table 2 displays the results of the simulation, ignoring transmittance and reflectance, with the setting power of 100 l m, the LED light intensity reached to the reagent was 9.05 l m after passing through the optical elements. Calculated with 95% transmittance, the light intensity was 7.76 l m after 3 optical elements. The fluorescence intensity was set to 1 l m and picked up by the detector with the intensity of 0.042 l m after optical path loss. Corresponding the above relationships to the actual parameters, the blue LED (LB W5SM) output at I = 350 mA was 15–33 l m, so the blue light projected to the reagent was 1.16–2.56 l m. Assumed with 65% fluorescence quantum efficiency of the reagent dye, the fluorescence intensity was 0.75–1.65 l m and the intensity reaching the detector was 0.031–0.069 l m. The PD (S1337-33BR, 5.7 cm^2^ detection area) had a current of 4.4–6.2 μA at 100 lx illumination, so the luminous flux on the detector surface at 100 lx was 5.7c×10^-4^ lm which was far less than 0.031 l m. Therefore, it can be considered that the fluorescence from samples was excited by the 470 nm excitation light, and then returned to the PD with very weak intensity which was still detectable despite significant losses.

**TABLE 2:** Results of the first channel simulation analysis.

	Light source	Light intensity (lm)	Receiving light intensity (lm)	With transmittance (95%) (lm)
Simulation	LED (470 nm)	100	9.05	7.76 (3 optical elements)
	Fluorescence (520 nm)	1	0.052	0.042 (4 optical elements)
Actual parameters	LB W5SM	15–33	1.36–2.99	1.16–2.56
	FAM	0.75–1.65	0.04–0.86	0.031–0.069

**FIGURE 7:**
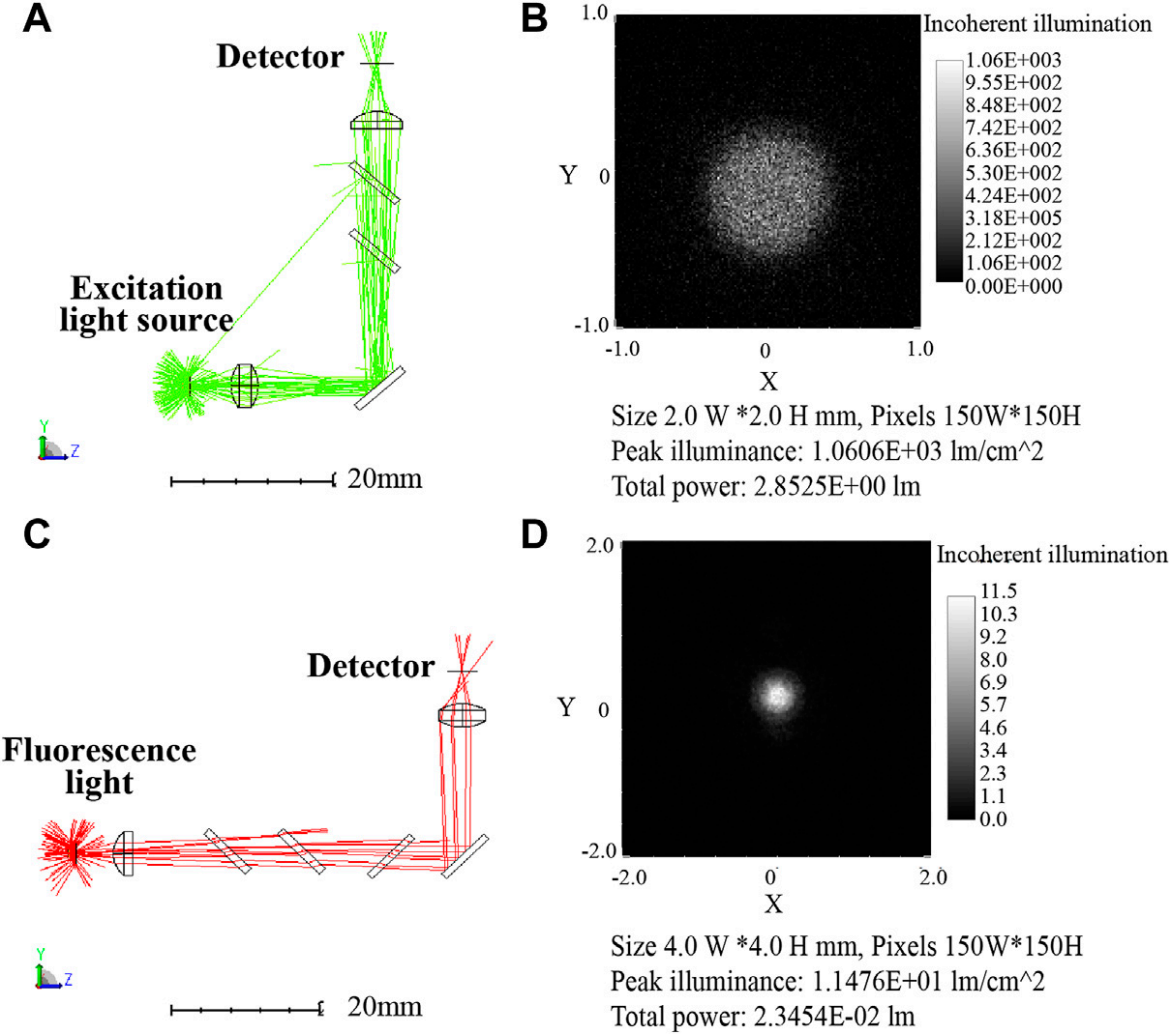
Second channel optical path (excitation/emission: 570 nm/630 nm) simulation diagram. **(A)** 3D layout of the excitation optical path with 2.85 l m total power. **(B)** Image of the excitation optical path detector. **(C)** 3D layout of the emission optical path. **(D)** Image of the emission optical path detector with 2.35 × 10^-2^ lm total power.

Similarly, second channel optical path simulation diagram is shown in Figure 7 and the its results are displayed in Table 3. The Yellow LED (LY W5SM) output was 39–82 l m and the fluorescence emitted from reagent was 0.56–1.18 l m. The light intensity received by the detector after six optical elements was 0.01–0.02 l m, much larger than 5.7 × 10^-4^ lm (the luminous flux on the PD at 100 lx). Although the second channel lost more light energy than the first, the faint fluorescence also can be detected by the PD, demonstrating the viability of the optical path.

**TABLE 3:** Results of the second channel simulation analysis.

	Light source	Light intensity (lm)	Receiving light intensity (lm)	With transmittance (95%)
				(lm)
Simulation	LED (570 nm)	100	2.85	2.21 (5 optical elements)
	Fluorescence (630 nm)	1	0.023	0.017 (6 optical elements)
Actual parameters	LY W5SM	39–82	1.11–2.34	0.86–1.81
	Texas Red	0.56–1.18	0.012–0.027	0.01–0.02

### HBV concentration gradient test

**FIGURE 8:**
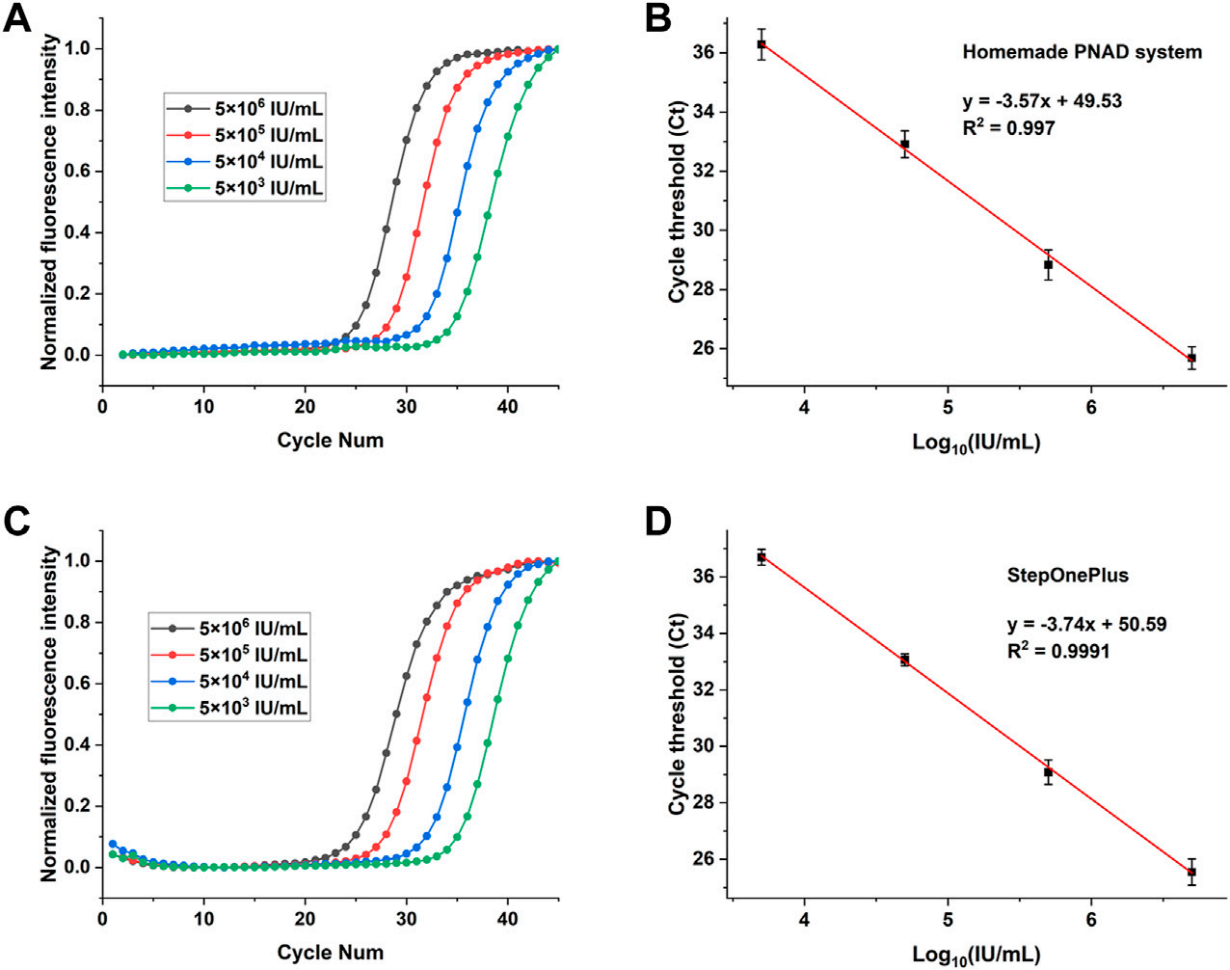
Real-time fluorescence PCR curves (left) and their standard curves (right) for detection of HBV with concentrations ranging from 5 × 10^6^ IU/ml to 5 × 10^3^ IU/ml. **(A)** and **(B)**: The real-time PCR curves and standard curves for Ct values from the homemade PNAD system. **(C,D)**: The fluorescence curves and standard curves for Ct values from StepOnePlus system.

Figure 8 shows the HBV concentration gradient test results from PNAD system and StepOnePlus. Real-time RT-PCR curves for HBV with concentration range from 5 × 10^6^ IU/ml to 5 × 10^3^ IU/ml are shown in Figures 8A,C, and standard curves for Ct values are shown in Figures 8B,D. In order to compare the results more visually, all original fluorescence data were baseline subtracted and normalized. In Figures 8A,C, the curves were aesthetically smooth and had the same trend for each concentration of HBV tested. Figures 8B,D indicates that standard curves from the PNAD system and StepOnePlus both had excellent linearity within the selected concentration range, and both had a determination coefficient *R* ^2^ > 0.99. Table 4 shows the results of Ct values and their reproducibility, the Ct values of HBV from the PNAD system and StepOnePlus were similar, and the CV of PNAD was within 2% which can be considered that the PNAD system equipped with this fluorescence module was fully comparable to the commercial StepOnePlus.

**TABLE 4:** Ct value and reproducibility of HBV concentration gradient test from the PNAD system and StepOnePlus system.

Concentration	PNAD system		StepOnePlus	
	Average ct value	CV(%)	Average ct value	CV(%)
5 × 10^6^ IU/ml	25.68	1.483	25.55	1.827
5 × 10^5^ IU/ml	28.93	1.756	29.08	1.495
5 × 10^4^ IU/ml	32.91	1.383	33.06	0.629
5 × 10^3^ IU/ml	36.29	1.876	36.69	0.762

### SARS-CoV-2 multi-target test

**FIGURE 9:**
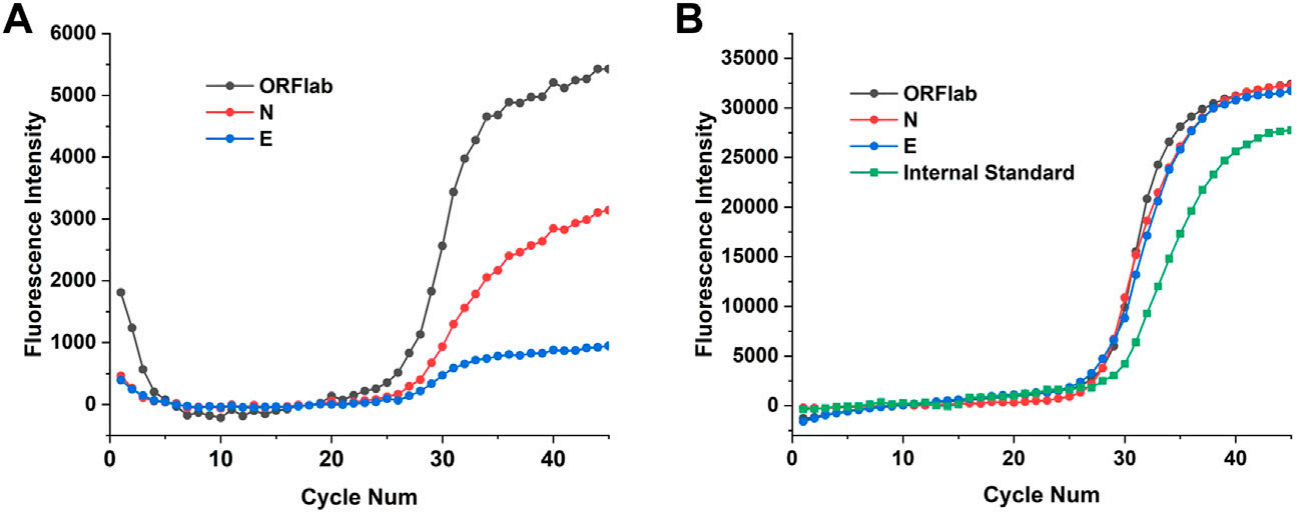
Multiplex real-time fluorescence PCR curves for detection of different targets of SARS-CoV-2. **(A)** Fluorescence curves of ORFlab gene, N gene and E gene of SARS-CoV-2 from StepOnePlus system. **(B)** Fluorescence curves of ORFlab gene, N gene, E gene and internal standard target from the homemade PNAD system.

To further validate performance of the 4 channels of the two fluorescence modules, the 2019-nCoV nucleic acid test kit was used to test ORFlab gene, N gene, E gene and the internal standard target of SARS-CoV-2. These four targets corresponding to the fluorescence channels are: FAM (470 nm/520 nm), HEX (520 nm/570 nm), TEXAS Red (570 nm/630 nm) and Cy5 (630 nm/690 nm), which coincided with the detection channels of the two fluorescence modules. Multiplex real-time fluorescence PCR curves for different genes of SARS-CoV-2 from StepOnePlus and the PNAD system are shown in the Figure 9. It should be noted that since there was no Cy 5 channel in StepOnePlus system, so the curve corresponding to the internal standard target was absence. Comparing with the curves from StepOnePlus system (Figure 9A), fluorescence curves from the PNAD system (Figure 9B) are smoother both in baseline and exponential growth periods, which means that under the driving of the current-light dual negative feedback LED circuit the excitation LED of the designed fluorescence module is highly stable. In addition, comparing with Figure 9A, the difference in signal gain of these curves was smaller in Figure 9B due to the compensation for optical loss by strengthening excitation light and amplifying fluorescence signal gain in the designed fluorescence module. The Ct values of each curves are displayed in Table 5, as for 3 different genes of SARS-CoV-2, the similar results of the PNAD system and StepOnePlus further indicate the homogeneity of each channels in fluorescence module.

**TABLE 5:** Ct value of different targets of SARS-CoV-2 from the PAND system and StepOnePlus system.

	Ct			
Target	ORFlab	N	E	Internal standard
StepOnePlus	27.83	27.97	27.86	\
PNAD System	27.67	28.12	27.82	30.32

## Conclusion

We developed a miniaturized, high-sensitivity and integrated dual-channel fluorescence module that was extremely suitable for portable nucleic acid detection system for real-time PCR. The dual-channel fluorescence module integrated drive circuit board and all of the optical elements in a minute (55 mm × 45 mm × 15 mm) metal box. Based on confocal optical path, a single module realized dual channels fluorescence exciting and detecting by integrating two set excitation-emission optical paths at different wavelengths. The stability of fluorescence signal is enhanced by the improvement of the LED stability which is provided by a current-light dual negative feedback LED circuit. In addition, the signal differences of each curves are smaller due to the compensation for optical loss by strengthening excitation light and amplifying fluorescence signal gain. After fabrication, we installed the two fluorescence modules on the homemade PNAD system to perform HBV concentration gradient test and multiplex test for different gene targets of SARS-CoV-2. The homemade PNAD system showed excellent linear response to HBV within the concentration range from 5 × 10^6^ IU/ml to 5 × 10^3^ IU/ml, the correlation coefficient *R* ^2^ was greater than 0.99. Besides, it had good reproducibility with CV less than 2%. A comparison of the SARS-CoV-2 test results between our modules and StepOnePlus shows that this module performs better in stability and homogeneity across different channels.

Currently, four dichroic mirrors are used in the optical path to distinguish excitation light from emission light, which greatly causes the loss of light energy. If more channels of fluorescence detection want to be achieved, the current optical path structure is not the most preferable solution. Because more detection channels mean more excitation sources, detectors and optical components such as dichroic mirrors and filters, there will be more losses in the optical path. So, we are considering the use of optical fiber to achieve the separation of excitation and emission light, which may reduce the loss of light energy. In addition to being integrated into a homemade PNAD system for real-time PCR, the fluorescence module designed in this paper can be adapted to different applications such as water quality monitoring, food safety checking and clinical testing by changing the LEDs and filters.

## Data Availability

The raw data supporting the conclusions of this article will be made available by the authors, without undue reservation.

## References

[B1] Abdallah Z. Rumeau A. Fernandez A. Cibiel G. Llopis O. (2014). Nonlinear equivalent-circuit modeling of a fast photodiode. IEEE Phot. Technol. Lett. 26 (18), 1840–1842. 10.1109/lpt.2014.2337352

[B2] Alam M. W. Wahid K. A. Goel R. K. Lukong K. E. (2019). Development of a low-cost and portable smart fluorometer for detecting breast cancer cells. Biomed. Opt. Express 10 (2), 399–410. 10.1364/BOE.10.000399 30800488 PMC6377908

[B3] Bedford J. Enria D. Giesecke J. Heymann D. L. Ihekweazu C. Kobinger G. (2020). COVID-19: Towards controlling of a pandemic. Lancet 395 (10229), 1015–1018. 10.1016/S0140-6736(20)30673-5 32197103 PMC7270596

[B4] Benmalek E. Elmhamdi J. Jilbab A. (2021). Comparing CT scan and chest X-ray imaging for COVID-19 diagnosis. Biomed. Eng. Adv. 1, 100003. 10.1016/j.bea.2021.100003 34786568 PMC7992299

[B5] Chen H. Wu Y. Chen Z. Hu Z. Fang Y. Liao P. (2017). Performance evaluation of a novel sample in-answer out (SIAO) system based on magnetic nanoparticles. J. Biomed. Nanotechnol. 13 (12), 1619–1630. 10.1166/jbn.2017.2478 29490751

[B6] Chen Z. Yang T. Yang H. Li T. Nie L. Mou X. (2018). A portable multi-channel turbidity system for rapid detection of pathogens by loop-mediated isothermal amplification. J. Biomed. Nanotechnol. 14 (1), 198–205. 10.1166/jbn.2018.2524 29463377

[B7] Cui F. Zhou H. S. (2020). Diagnostic methods and potential portable biosensors for coronavirus disease 2019. Biosens. Bioelectron. X. 165, 112349. 10.1016/j.bios.2020.112349 PMC7266610 32510340

[B8] Deng H. Jayawardena A. Chan J. Tan S. M. Alan T. Kwan P. (2021). An ultra-portable, self-contained point-of-care nucleic acid amplification test for diagnosis of active COVID-19 infection. Sci. Rep. 11 (1), 15176. 10.1038/s41598-021-94652-0 34312441 PMC8313664

[B9] Fang Y. Liu H. Wang Y. Su X. Jin L. Wu Y. (2021). Fast and accurate control strategy for portable nucleic acid detection (PNAD) system based on magnetic nanoparticles. J. Biomed. Nanotechnol. 17 (3), 407–415. 10.1166/jbn.2021.3028 33875075

[B10] Fu L. Qian Y. Zhou J. Zheng L. Wang Y. (2020). Fluorescence-based quantitative platform for ultrasensitive food allergen detection: From immunoassays to DNA sensors. Compr. Rev. Food Sci. Food Saf. 19 (6), 3343–3364. 10.1111/1541-4337.12641 33337031

[B11] Hanson K. E. Azar M. M. Banerjee R. Chou A. Colgrove R. C. Ginocchio C. C. (2020). Molecular testing for acute respiratory tract infections: Clinical and diagnostic recommendations from the IDSA’s diagnostics committee. Clin. Infect. Dis. 71 (10), 2744–2751. 10.1093/cid/ciaa508 32369578 PMC7454374

[B12] Huang L. Su E. Liu Y. He N. Deng Y. Jin L. (2021). A microfluidic device for accurate detection of hs-cTnI. Chin. Chem. Lett. 32 (4), 1555–1558. 10.1016/j.cclet.2020.09.055

[B13] Ishikawa-Ankerhold H. C. Ankerhold R. Drummen G. P. (2012). Advanced fluorescence microscopy techniques--FRAP, FLIP, FLAP, FRET and FLIM. Molecules 17 (4), 4047–4132. 10.3390/molecules17044047 22469598 PMC6268795

[B14] Katzmeier F. Aufinger L. Dupin A. Quintero J. Lenz M. Bauer L. (2019). A low-cost fluorescence reader for *in vitro* transcription and nucleic acid detection with Cas13a. PLoS One 14 (12), e0220091. 10.1371/journal.pone.0220091 31851676 PMC6919979

[B15] Li C. Ren L. (2020). Recent progress on the diagnosis of 2019 novel coronavirus. Transbound. Emerg. Dis. 67 (4), 1485–1491. 10.1111/tbed.13620 32395897 PMC7272792

[B16] Mathuria J. P. Yadav R. Rajkumar Y. (2020). Laboratory diagnosis of SARS-CoV-2 - a review of current methods. J. Infect. public health 13 (7), 901–905. 10.1016/j.jiph.2020.06.005 32534946 PMC7275982

[B17] Novak L. Neuzil P. Pipper J. Zhang Y. Lee S. (2007). An integrated fluorescence detection system for lab-on-a-chip applications. Lab. Chip 7 (1), 27–29. 10.1039/b611745g 17180202

[B18] Peng Y. M. Pan J. Z. Fang Q. (2021). Handheld laser-induced fluorescence detection systems with different optical configurations. Talanta 230, 122329. 10.1016/j.talanta.2021.122329 33934786

[B19] Pokhrel P. Hu C. Mao H. (2020). Detecting the coronavirus (COVID-19). ACS Sens. 5 (8), 2283–2296. 10.1021/acssensors.0c01153 32627534 PMC7366507

[B20] Rai P. Kumar B. K. Deekshit V. K. Karunasagar I. Karunasagar I. (2021). Detection technologies and recent developments in the diagnosis of COVID-19 infection. Appl. Microbiol. Biotechnol. 105 (2), 441–455. 10.1007/s00253-020-11061-5 33394144 PMC7780074

[B21] Ryu G. Huang J. Hofmann O. Walshe C. A. Sze J. Y. Y. McClean G. D. (2011). Highly sensitive fluorescence detection system for microfluidic lab-on-a-chip. Lab. Chip 11 (9), 1664. 10.1039/c0lc00586j 21431240

[B22] Shin Y.-H. Teresa Gutierrez-Wing M. Choi J.-W. (2021). Review—recent progress in portable fluorescence sensors. J. Electrochem. Soc. 168 (1), 017502. 10.1149/1945-7111/abd494

[B23] Song Q. Sun X. Dai Z. Gao Y. Gong X. Zhou B. (2021). Point-of-care testing detection methods for COVID-19. Lab. Chip 21 (9), 1634–1660. 10.1039/d0lc01156h 33705507

[B24] Song T. Zhang B. Luo Q. Q. (2010). Photoeletric conversion circuit design and optimization. Electro-Opt. Technol. Appl. 25 (06), 46–48.

[B25] Spibey C. A. Jackson P. Herick K. (2001). A unique charge-coupled device/xenon arc lamp based imaging system for the accurate detection and quantitation of multicolour fluorescence. Electrophoresis 22 (5), 829–836. 10.1002/1522-2683()22:5<829::AID-ELPS829>3.0.CO;2-U 11332749

[B26] Tang C. He Z. Liu H. Xu Y. Huang H. Yang G. (2020). Application of magnetic nanoparticles in nucleic acid detection. J. Nanobiotechnology 18 (1), 62. 10.1186/s12951-020-00613-6 32316985 PMC7171821

[B27] Wang C. Liu M. Wang Z. Li S. Deng Y. He N. (2021). Point-of-care diagnostics for infectious diseases: From methods to devices. Nano Today 37, 101092. 10.1016/j.nantod.2021.101092 33584847 PMC7864790

[B28] Xie X. R. Yang B. (2019). Optical system design of multichannel PCR fluorescene detector. Opt. Tech. 45 (5), 531–534. 10.13741/j.cnki.11-1879/o4.2019.05.004

[B29] Zhan X. Liu Q. Wang Y. Tian H. Hu A. He X. (2019). Coupled equivalent circuit for high-speed photodiodes. IEEE Electron Device Lett. 40 (10), 1654–1657. 10.1109/led.2019.2937677

[B30] Zhu H. Zhang H. Ni S. Korabecna M. Yobas L. Neuzil P. (2020). The vision of point-of-care PCR tests for the COVID-19 pandemic and beyond. TrAC Trends Anal. Chem. 130, 115984. 10.1016/j.trac.2020.115984 PMC7369599 32834243

